# Impact of the Family and Household Environment on Pediatric Atopic Dermatitis in Japan

**DOI:** 10.3390/jcm12082988

**Published:** 2023-04-20

**Authors:** Hidehisa Saeki, Yukihiro Ohya, Hisakatsu Nawata, Kazuhiko Arima, Miho Inukai, Ana B. Rossi, Gaelle Bego-Le-Bagousse

**Affiliations:** 1 Department of Dermatology, Nippon Medical School, 1-1-5 Sendagi, Bunkyo-ku, Tokyo 113-8603, Japan; 2 Allergy Center, National Center for Child Health and Development, Setagaya-ku, Tokyo 157-8535, Japan; 3 Immunology Medical, Sanofi K.K., Shinjuku-ku, Tokyo 163-1488, Japan; 4 Market Access, Sanofi K.K., Shinjuku-ku, Tokyo 163-1488, Japan; 5 Sanofi, Cambridge, MA 02139, USA; 6 Sanofi, Chilly-Mazarin, 91380 Paris, France

**Keywords:** atopic, child, dermatitis, family, quality of life

## Abstract

Pediatric atopic dermatitis (AD) can negatively impact the family quality of life (QoL). We report data from the real-world Epidemiology of Children with Atopic Dermatitis Reporting on their Experience (EPI-CARE) study in Japanese pediatric patients, focusing on disease impact on family QoL. Children and adolescents aged 6 months to <18 years completed an online survey between September 2018–December 2019. The impact of disease severity on family QoL and its effect on parents’ time were assessed using the dermatitis family impact (DFI) questionnaire. The impact of a family history of allergic conditions, current residency, second-hand smoke exposure, and household pets on AD prevalence and severity was also assessed. Family QoL decreased as AD severity increased, particularly in families with children aged <6 years; but had the greatest impact on sleep and tiredness in families with children aged <12 years. Parents spent at least 4.6 h/week caring for children <6 years, including those with mild symptoms. Most children (>80%) had a family history of allergic conditions; AD prevalence was increased in those exposed to second-hand smoke or household pets. This study demonstrated that pediatric AD in Japanese individuals has negative impacts on family QoL and that family and household environments can influence pediatric AD prevalence.

## 1. Introduction

Atopic dermatitis (AD) is a chronic, relapsing skin condition characterized by pruritus and disruption of the epithelial barrier, with symptoms often appearing within the first 5 years of life and persisting into adolescence and adulthood [1,2].

The pathophysiology of AD involves a complex interaction between genetic epithelial skin barrier defects, dysregulated immune response, an altered microbiome, and environmental risk factors [3]. In particular, patients with atopic diathesis are genetically predisposed to develop one or more atopic diseases, including AD, hay fever, allergic rhinitis, and asthma [4]. In addition, the patient’s exposome, a concept that encompasses all environmental exposure (e.g., chemical pollution, tobacco smoke, infectious agents, or lifestyle factors) encountered by an individual throughout their life, is thought to play an important role in AD development [5,6,7,8,9].

AD in childhood and adolescence has a negative impact on the quality of life (QoL) of the parents and caregivers of affected children [10,11,12,13]. Moderate-to-severe AD in children is often associated with a significant symptom burden, including pruritus, pain, and sleep disturbance, which may result in poor self-esteem, reduced school performance, and increased familial stress [14]. Parents of children with AD often report reduced QoL related to sleep disturbances, daytime functioning, and financial problems [10,11,12]. A multinational survey on the impact of AD on family QoL reported that caring for a child with moderate-to-severe AD was associated with high levels of parental anxiety and exhaustion, as well as strained family relationships [10]. Life decisions for caregivers and families of children with AD are also negatively impacted, particularly in children with severe disease [15].

In addition to the negative impact of AD on families, the prevalence or severity of AD may be affected by several factors, including a family history of allergic conditions [16], second-hand smoke exposure [17], and the presence of domestic pets [18]. However, few studies have evaluated the impact of pediatric AD on family QoL or the impact of the family and household environment on AD prevalence and severity in Japan.

The Epidemiology of Children with Atopic Dermatitis Reporting on their Experience (EPI-CARE) study evaluated the prevalence and burden of AD among the pediatric population (aged 6 months to <18 years) and their caregivers in countries from several geographic regions worldwide, including Japan [19]. Data from the EPI-CARE study have indicated that parents and caregivers experience an impact of their child’s AD on family QoL, particularly in children with higher disease severity [13]. In the Japanese EPI-CARE pediatric population (n = 5702), the 12-month prevalence of diagnosed AD (based on meeting the International Study of Asthma and Allergies in Childhood (ISAAC) criteria [20] and having a physician-diagnosed AD) was 10.7% overall, 12.9% in patients aged <6 years, 10.3% in those aged 6 to <12 years, and 9.1% in those aged 12 to <18 years [19].

Here, we report additional results of the EPI-CARE study in the Japanese pediatric population, focusing on the impact of pediatric AD on family QoL and the influence of the family and the household environment on its prevalence and severity.

## 2. Materials and Methods

### 2.1. Study Design

The design of the cross-sectional, epidemiologic EPI-CARE study has been described previously [19]. In addition, EPI-CARE was conducted across 18 countries to collect representative data from pediatric populations with AD. The study received ethical approval and was performed in accordance with the European Union General Data Protection Regulation, the European Society for Opinion and Marketing Research, the Insights Association, the European Pharmaceutical Marketing Research Association, the British Healthcare Business Intelligence Association, the US Health Insurance Portability and Accountability Act, and all international and local data protection legislation. All participants or their parents provided written informed consent before study entry. No personally identifiable information or medical data were collected.

A web-based survey was used for data collection, and the parents of participants were recruited via direct emailing, special interest websites, and broad-reach portals. Kantar Health was responsible for participant recruitment, administration of surveys, collation of responses, and data analyses. The survey was conducted in Japan between 26 September 2018, and 2 December 2019.

### 2.2. Study Population

The study included children and adolescents aged 6 months to <18 years. There were no other specific study inclusion/exclusion criteria.

Initially, parents of eligible children and adolescents were recruited via email and participated in an online panel; panel members were blinded to the research topic when invited and received points once they completed the survey that could be redeemed for items in a prize catalog (exact values unknown). After initial recruitment, the parents of children aged <12 years completed the survey on behalf of their children, and adolescents aged 12 to <18 years completed the survey themselves.

### 2.3. Questionnaire and Outcomes

The 30-min online, web-based survey included two sections. In the first section, a selection algorithm was used to determine which children were to be investigated (in cases where parents had multiple children), and demographic data were collected. Three definitions of AD were used: ‘reported AD,’ ‘physician-diagnosed AD,’ and ‘diagnosed AD’. For ‘reported AD’ responders had to exclusively meet all three ISAAC criteria (Table 1) [20]. For ‘physician-diagnosed AD’ responders self-reported having ever been told by a physician that they had AD. For ‘diagnosed AD’ responders met all three ISAAC criteria and had self-reported having ever been told by a physician that they had AD.

In the second section of the survey, which was completed by responders with AD, disease severity was evaluated, and data on the impact of family and home life on the disease burden on the individual’s family were collected. AD severity in the past week was evaluated by the patient-oriented eczema measure (POEM) [21], with total scores ranging from 0 (lowest severity) to 28 (highest severity). POEM scores of 0 to 7 indicate mild disease, 8 to 16 indicate moderate disease, and >16 indicate severe disease [22]. Disease severity was also assessed using the patient global assessment (PtGA) [23], with self-reported severity classified as clear/mild, moderate, or severe.

The impact of disease burden on the QoL of the parents and family of the individual with AD was assessed using the dermatitis family impact (DFI) questionnaire [24]. The DFI questionnaire assesses the impact of disease on (1) housework, (2) food preparation and feeding, (3) sleep of other family members, (4) family leisure activities, (5) time spent shopping for the family, (6) expenditure, (7) causing tiredness and exhaustion of parents/caregivers, (8) causing emotional distress of parents/caregivers, (9) relationships between the main caregiver and partner or other children, and (10) the main caregiver’s life. Total scores range from 0 to 30, with higher scores indicating a greater impact on family life [24]. The impact of the disease on the time spent caring for the child and the number of workdays missed was also examined.

Additionally, the impact of a family history of allergic conditions, including AD, hay fever, or asthma, on AD severity was assessed. The impact of second-hand smoke exposure and the presence of domestic pets on the prevalence and severity of AD was also examined.

### 2.4. Statistical Analysis

The target population size was determined prior to data collection to ensure that the surveyed individuals were representative of the population in Japan for sex, age, geographic regions, and urban versus rural residence. A weighting adjustment was applied if this target was not met exactly, as previously described [19].

Descriptive statistics were used to present the data, with continuous data described by the mean, median, standard deviation (SD), and range. The numbers of individuals as a proportion of the sample, means, and medians were weighted, while the absolute numbers of individuals were unweighted.

## 3. Results

### 3.1. Study Population Diagnosed with AD

Of the 5702 pediatric patients in the Japanese EPI-CARE population, 1671 (29.3%) were aged 6 months to <6 years, 1989 (34.9%) were aged 6 to <12 years and 2042 (35.8%) were aged 12 to <18 years. The prevalence of diagnosed AD was 12.9% (n = 226) in children aged <6 years, 10.3% (n = 200) in children aged 6 to <12 years, and 9.1% (n = 182) among adolescents aged 12 to <18 years.

The proportion of patients with clear/mild AD, moderate AD, and severe AD based on the POEM score was 63.2%, 32.4%, and 4.4%, respectively, and based on PtGA scores, it was 72.3%, 25.5%, and 2.2%, respectively [19].

### 3.2. Impact on Family QoL

**Figure 1:**
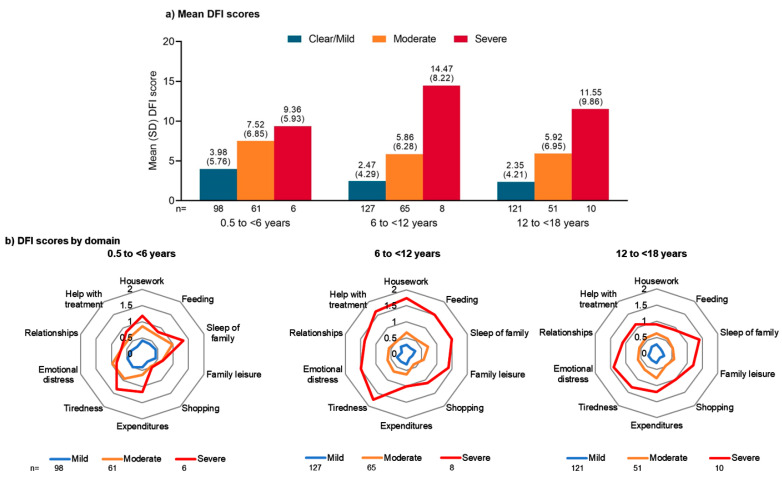
(**a**) Mean dermatitis family impact (DFI) scores according to POEM severity of atopic dermatitis in Japanese pediatric patients across age groups; (**b**) distribution of DFI domain scores across age groups. DFI, dermatitis family impact; POEM, patient-orientated eczema measure; SD, standard deviation.

In all age groups, mean DFI scores increased as the severity of ‘diagnosed AD’ increased (Figure 1a). In patients with clear/mild AD based on POEM scores, mean (SD) DFI scores were numerically higher in children aged <6 years than in those aged 6 to <12 years or 12 to <18 years (3.98 (5.76) vs 2.47 (4.29) and 2.35 (4.21), respectively). The mean (SD) DFI scores were also numerically higher in children with moderate AD aged <6 years (7.52 (6.85)) than those aged 6 to <12 years (5.86 (6.28)) or 12 to <18 years (5.92 (6.95)). Among patients with severe AD, mean (SD) DFI scores were 9.36 (5.93) in children aged <6 years, 14.47 (8.22) in those aged 6 to <12 years, and 11.55 (9.86) in adolescents aged 12 to <18 years.

When DFI scores were examined by individual domains in children aged <6 years, increased disease severity had the greatest impact on the ‘sleep of family’ and ‘tiredness’ domains (Figure 1b). In children aged 6 to <12 years, severe AD had a marked impact on all DFI domains, with the greatest impact being on ‘tiredness.’ In adolescents aged 12 to <18 years, moderate AD had the greatest impact on the ‘expenditures’ domain, and severe AD had the greatest impact on the ‘emotional distress,’ ‘sleep of family,’ and ‘tiredness’ domains.

### 3.3. Impact on Parents’ Time

**Figure 2:**
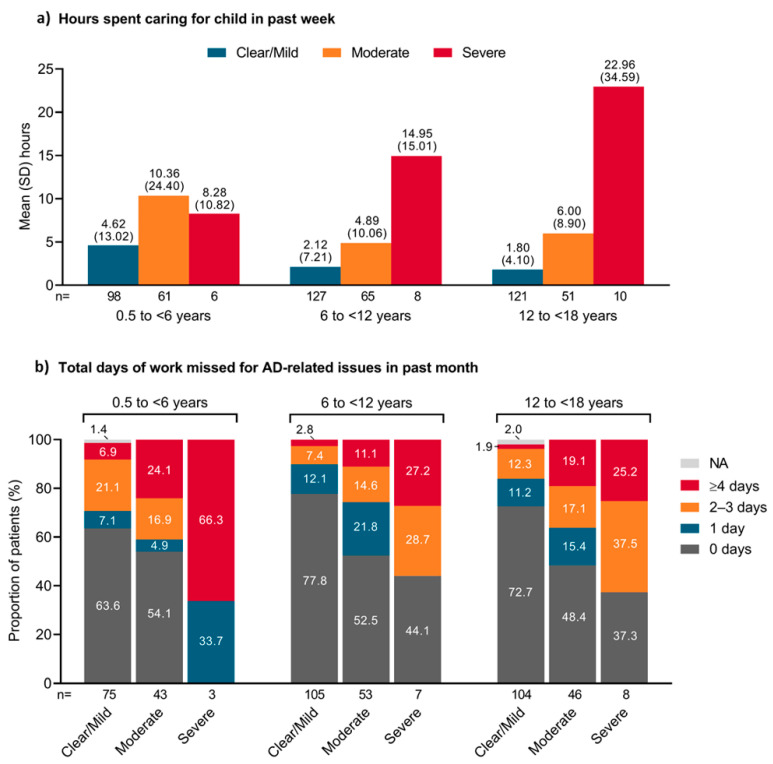
(**a**) Mean hours spent caring for child in the past week; (**b**) total days of work missed for atopic dermatitis-related issues in the past month for parents/caregivers of Japanese pediatric patients across age groups. AD, atopic dermatitis; NA, not applicable; SD, standard deviation.

In general, the mean number of hours spent caring for a child with ‘diagnosed AD’ in the past week increased as AD severity increased (Figure 2a). In children aged <6 years, a mean of 4.62 h was spent by parents caring for those who had mild diseases. In all age groups, the length of time spent caring for those with moderate or severe AD was more than twice that needed for those with mild AD. In patients aged 6 to <12 years and 12 to <18 years, the time needed to care for those with severe AD was more than three times that required for those with moderate AD.

The number of workdays missed for AD-related issues in the past month also increased as AD severity increased (Figure 2b). Among parents of children or adolescents with severe AD, at least 1 workday was missed by 100.0% of parents of children aged <6 years, 55.9% of those aged 6 to <12 years, and 62.7% of those aged 12 to <18 years.

### 3.4. Family History of Allergic Conditions

**Figure 3:**
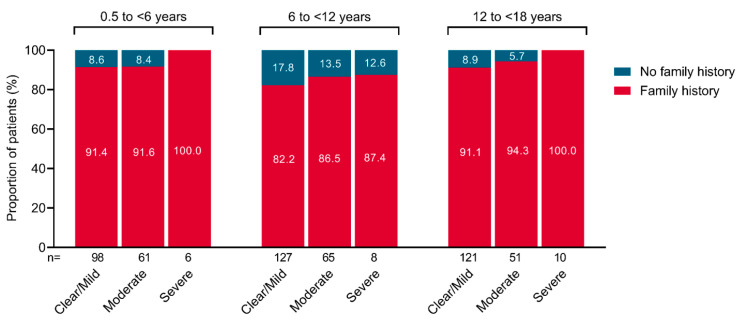
Proportion of patients with a family history of atopic dermatitis (AD), hay fever, or asthma according to POEM severity of AD in Japanese pediatric patients across age groups. POEM, patient-orientated eczema measure.

There was a family history of AD, hay fever, or asthma in 488/547 patients (89.1%) with ‘diagnosed AD’. In children aged <6 years, 91.4% of those with clear/mild AD, 91.6% of those with moderate disease, and 100.0% of those with severe disease had a family history of AD, hay fever, or asthma (Figure 3). Similar trends were observed in patients aged 6 to <12 years and 12 to <18 years.

### 3.5. Current Residency

The majority of pediatric patients with AD lived in urban or suburban regions of the country across all age groups, regardless of AD severity (Supplementary Figure S1). Current residency did not appear to impact disease severity in any age group.

### 3.6. Second-Hand Smoke Exposure

**Figure 4:**
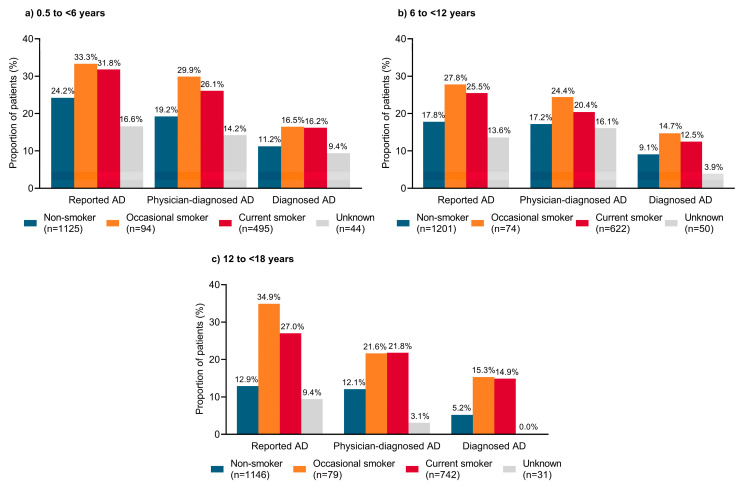
Prevalence of atopic dermatitis according to family smoking status in Japanese pediatric patients aged (**a**) 0.5 to <6 years; (**b**) 6 to <12 years; (**c**) 12 to <18 years. AD, atopic dermatitis.

The prevalence of ‘reported AD,’ ‘physician-diagnosed AD,’ and ‘diagnosed AD’ was higher in children or adolescents with a current or occasional smoker in the household than in those from a non-smoking household (Figure 4). Across age groups, the prevalence of ‘reported AD’ ranged from 12.9–24.2% in individuals living with non-smokers, from 25.5–31.8% in those living with a current smoker, and from 27.8–33.3% in those living with an occasional smoker. In young children aged <6 years, 83.0% of those with severe ‘diagnosed AD’ were living with a smoker in the household (Supplementary Figure S2).

### 3.7. Household Pets

**Figure 5:**
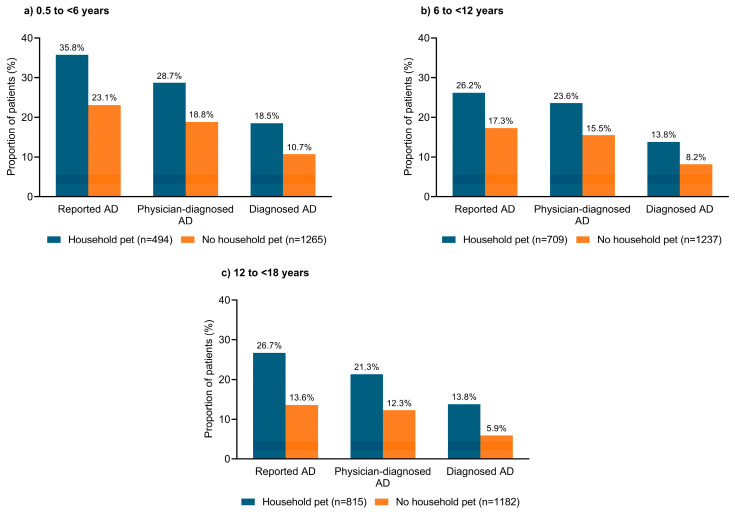
Prevalence of atopic dermatitis according to the presence of a household pet in Japanese pediatric patients aged (**a**) 0.5 to <6 years; (**b**) 6 to <12 years; (**c**) 12 to <18 years. AD, atopic dermatitis.

The prevalence of ‘reported AD,’ ‘physician-diagnosed AD,’ and ‘diagnosed AD’ was higher among children or adolescents who lived with a household pet than in those without a household pet (Figure 5). Among those living with a household pet, 26.2–35.8% had ‘reported AD,’ 21.3–28.7% had ‘physician-diagnosed AD,’ and 13.8–18.5% had ‘diagnosed AD’. In contrast, in children living without a household pet, 13–23.1% had ‘reported AD,’ 12.3–18.8% had ‘physician-diagnosed AD,’ and 5.9–10.7% had ‘diagnosed AD’. However, the number of household pets did not appear to impact disease severity in any of the age groups (Supplementary Figure S3).

### 3.8. Parent Education and Employment Status

In general, a high proportion of the parents of affected children/adolescents had college, university, or graduate school education across all age groups, regardless of AD severity (Supplementary Figure S4). In addition, the majority of parents were employed (Supplementary Figure S5). Education and employment status did not appear to impact disease severity in any age group.

## 4. Discussion

In this Japanese pediatric population of the EPI-CARE study, 10.7% of patients had been diagnosed with AD within the last 12 months [19]. According to the POEM tool, the majority of those with diagnosed AD had a clear/mild disease (63.2%), while 32.4% and 4.4% had moderate or severe AD, respectively [19].

In the current analysis, the DFI questionnaire showed that family QoL decreased as the severity of their child’s AD increased. The mean DFI scores ranged from 2.35–3.98 across age groups in patients with clear/mild AD, from 5.86–7.52 in those with moderate AD, and from 9.36–14.47 in those with severe AD. In patients with clear/mild, or moderate AD, mean DFI scores were numerically higher for families with children aged <6 years than for families with older children. When the individual DFI domains were evaluated, disease severity appeared to have the greatest impact on the ‘sleep of family’ and ‘tiredness’ among families with children aged <6 years or 6 to <12 years.

These results are consistent with those of an international web survey [10] and the Avon Longitudinal Study of Parents and Children (ALSPAC) study in the UK [25]. In the international web survey (n = 235), parental sleep disturbance and fatigue both increased as the child’s AD severity increased [10]. Similarly, the ALSPAC study, which included 11,649 mother–child pairs, showed that mothers of children with mild, moderate, or severe AD had difficulty falling asleep, subjectively insufficient sleep, and daytime exhaustion during the first 11 years of their child’s life [25]. In Japanese families, young children and their parents often sleep in the same room, so parents will often experience sleep disturbances if their child has sleeping difficulties due to AD symptoms.

The current analysis showed a marked decrease in family QoL among children aged 6 to <12 years with severe AD, most likely because parents are solely responsible for managing skin care in this age group. Our results also showed that the burden on parents and families is greater in children aged <6 years than in those aged 6 to <12 years or 12 to <18 years for mild or moderate AD. This may be because younger children with AD are less able to cope with their symptoms and require more care from their parents/caregivers than older children or adolescents.

The time spent by parents caring for their children with AD in the past week increased in proportion to disease severity in children aged ≥6 years, being more than threefold higher for parents with children/adolescents with severe AD than for those with moderate AD. In children aged <6 years, parents spent an average of at least 4.6 h per week caring for their children, even those with mild symptoms. The increase in the time required to care for children aged ≥6 years was associated with an increase in the number of workdays missed by parents for AD-related issues in the past month, particularly when their child had severe AD. The increase in work absences may be caused by the child being absent from school. These findings are in line with those of a cross-sectional United States study that included 3132 children with AD and found a two-fold higher likelihood of chronic school absenteeism (i.e., ≥15 days missed per year) among children with severe AD versus mild-to-moderate AD. The same study found a significantly higher number of workdays missed among both fathers (*p* = 0.03) and mothers (*p* < 0.0001) of children with AD [26].

Among patients with ‘diagnosed AD,’ most (89.1%) had a family history of AD, hay fever, or asthma. The proportion of patients with a family history of allergic conditions was >80% across all age groups and all disease severity classifications, suggesting that a family history of allergies plays a role in the development of AD at any age, regardless of disease severity. This is consistent with the concept of atopic diathesis, whereby atopic diseases such as AD, asthma, and allergic rhinitis are genetically linked [4]. Data from the ALSPAC study indicated that a parental history of AD was a strong predictor of AD in their offspring, although a history of asthma or hay fever alone was only associated with childhood AD if both parents had these conditions [16]. In the Japan Environment and Children’s Study, the lifetime prevalence of parental AD was 15.7% among mothers and 11.2% among fathers [27].

A correlation between second-hand smoke exposure, AD development, and AD severity has been observed in several studies, including a meta-analysis [17], Japanese studies [28,29,30], and studies from other countries [31,32,33]. In a Japanese survey of 4466 adolescents aged 13–14 years, household smoking was an important modifiable risk factor for AD disease severity [29]. In a cross-sectional Japanese study of 1177 parent–infant pairs, fetal smoke exposure after 28 weeks gestation was associated with significantly higher adjusted odds of AD syndrome in infants aged >6 months compared with unexposed infants (adjusted odds ratio (aOR), 5.21; 95% confidence interval (CI), 1.08–25.15; *p* = 0.020) [28]. Similarly, a Japanese prospective pre-birth cohort study of 1354 mother–child pairs showed higher adjusted odds of physician-diagnosed AD in children with prenatal smoking exposure compared with no prenatal smoking exposure (aOR, 7.11; 95% CI, 1.43–27.8). However, there was no association between perinatal smoking exposure and AD defined according to ISAAC criteria [30]. In a meta-analysis of 86 studies from 39 countries, second-hand smoke exposure was associated with increased odds of AD in children aged <18 years (OR, 1.18; 95% CI, 1.01–1.38), but the odds of AD in childhood were not significantly increased with maternal smoking exposure during pregnancy [17]. The findings of the current analysis were generally in line with previous studies, with an increased prevalence of ‘reported AD,’ ‘physician-diagnosed AD,’ and ‘diagnosed AD’ among children or adolescents living with a current or occasional smoker compared with those living in a non-smoking household. However, statistical significance testing and logistic regression analyses were not conducted. In contrast, the Osaka Maternal and Child Health Study in 865 Japanese parent–child pairs showed that maternal smoking was not related to an increased risk of suspected AD [34].

Randomized studies on the association between household pets and pediatric AD are difficult to conduct, and data from observational studies are conflicting. A previous study of 3864 school children has shown a statistical association between having a pet rabbit and severe AD (adjusted prevalence ratio (aPR), 1.94; 95% CI, 1.02–3.71) or study-defined current AD (defined as physician-diagnosed AD and/or ever having a recurrent itchy rash for ≥6 months and having a current itchy flexural rash; aPR, 1.45; 95% CI, 1.07–1.20), and between having a pet cat and physician-diagnosed AD (aPR, 1.25; 95% CI, 1.03–1.50). In contrast, having a pet dog was not statistically associated with AD symptoms [18]. Alternatively, the Osaka Maternal and Child Health study found that exposure to indoor domestic pets (i.e., dogs, cats, birds, or hamsters) during pregnancy was not statistically associated with AD in 865 infants (aOR, 1.15; 95% CI, 0.55–2.25) [34]. Another Japanese study, which surveyed 35,242 schoolchildren aged 6 years, reported that cat ownership was associated with a significantly lower prevalence of AD (aOR, 0.79; 95% CI, 0.67–0.93) [35]. In the current analysis, AD prevalence appeared to be higher among children or adolescents who lived with a household pet than in those without pets, although there was no association between household pets and disease severity. The Japanese guidelines for AD recommend avoiding pets in households with individuals who test positive for specific immunoglobulin E antibodies in animals (e.g., dogs, cats, other mammals, birds, and hamsters) [36].

The apparent increase in AD prevalence with second-hand smoke exposure and household pets in the current study is consistent with the concept of the exposome, whereby environmental exposure to various factors leads to disruption of the epithelial skin and mucosal barriers and subsequent development of atopic diseases such as AD, allergic rhinitis, food allergies, chronic rhinosinusitis, and asthma [5,6,7,8,9]. In this context, aryl hydrocarbon receptor (AhR) signaling pathways are thought to be involved in regulating skin homeostasis in response to environmental exposure, which may have therapeutic implications for the pharmacologic management of AD [37,38].

The limitations of this study include its retrospective design, the small number of individuals with severe AD and the lack of long-term follow-up, sampling and nonresponse bias, potential recall bias due to the survey being completed by the parents of children aged <12 years or by adolescents aged 12 to <18 years, and the lack of statistical significance or regression analyses. In addition, the data were collected from Japanese patients only, which may limit the generalizability of these results to other ethnicities. However, the strengths of this study include the collection of country-specific representative data and the use of ISAAC criteria to identify individuals with reported or diagnosed AD, which allows for a consistent method of evaluating AD prevalence.

## 5. Conclusions

In conclusion, this population-based study of Japanese children and adolescents showed that AD had a negative impact on family QoL, especially the sleep of family members, tiredness, and the time spent caring for the child, which increased with increasing disease severity. A family history of allergic conditions was present in >80% of children or adolescents with AD, and the prevalence of AD in the past 12 months appeared to be higher among children exposed to second-hand smoke and in those living with a household pet.

## Figures and Tables

**Table 1:** International Study of Asthma and Allergies in Childhood Criteria for atopic dermatitis [20].

Criteria	Description
1	An itchy rash that has come and gone for ≥6 months
2	The itchy rash has appeared at any time in the past 12 months
3	The itchy rash has affected any of the following places at any time: Folds of the elbows Behind the knees In front of the ankles Under the buttocks Around the neck, ears, or eyes

## Data Availability

The datasets generated/analyzed during the current study are available from the corresponding author upon reasonable request.

## Supplementary Materials

The following supporting information can be downloaded at: https://www.mdpi.com/article/10.3390/jcm12082988/s1, Figure S1: Current residency of families of Japanese pediatric patients with atopic dermatitis by disease severity and age group; Figure S2: Proportion of households with or without smokers by the severity of atopic dermatitis in Japanese pediatric patients across age groups; Figure S3: Number of household pets by the severity of atopic dermatitis in Japanese pediatric patients across age groups; Figure S4: Parents’ highest education level by the severity of atopic dermatitis in Japanese pediatric patients across age groups; Figure S5: Parents’ employment status by the severity of atopic dermatitis in Japanese pediatric patients across age groups.

## References

[1] Irvine A.D., Mina-Osorio P. (2019). Disease trajectories in childhood atopic dermatitis: An update and practitioner’s guide. Br. J. Dermatol..

[2] Lyons J.J., Milner J.D., Stone K.D. (2015). Atopic dermatitis in children: Clinical features, pathophysiology, and treatment. Immunol. Allergy Clin. N. Am..

[3] Napolitano M., Fabbrocini G., Martora F., Genco L., Noto M., Patruno C. (2022). Children atopic dermatitis: Diagnosis, mimics, overlaps, and therapeutic implication. Dermatol. Ther..

[4] Novak N., Bieber T. (2003). Allergic and nonallergic forms of atopic diseases. J. Allergy Clin. Immunol..

[5] Celebi Sozener Z., Özbey Yücel Ü., Altiner S., Ozdel Oztürk B., Cerci P., Türk M., Gorgülü Akin B., Akdis M., Yilmaz I., Ozdemir C. (2022). The external exposome and allergies: From the perspective of the epithelial barrier hypothesis. Front. Allergy.

[6] Molina-García M., Granger C., Trullàs C., Puig S. (2022). Exposome and skin: Part 1. Bibliometric analysis and review of the impact of exposome approaches on dermatology. Dermatol. Ther..

[7] Stefanovic N., Flohr C., Irvine A.D. (2020). The exposome in atopic dermatitis. Allergy.

[8] Stefanovic N., Irvine A.D., Flohr C. (2021). The role of the environment and exposome in atopic dermatitis. Curr. Treat. Options Allergy.

[9] Celebi Sozener Z., Ozdel Ozturk B., Cerci P., Turk M., Gorgulu Akin B., Akdis M., Altiner S., Ozbey U., Ogulur I., Mitamura Y. (2022). Epithelial barrier hypothesis: Effect of the external exposome on the microbiome and epithelial barriers in allergic disease. Allergy.

[10] Capozza K., Gadd H., Kelley K., Russell S., Shi V., Schwartz A. (2020). Insights from caregivers on the impact of pediatric atopic dermatitis on families: “I’m tired, overwhelmed, and feel like I’m failing as a mother”. Dermatitis.

[11] Meltzer L.J., Flewelling K.D., Jump S., Gyorkos E., White M., Hauk P.J. (2020). Impact of atopic dermatitis treatment on child and parent sleep, daytime functioning, and quality of life. Ann. Allergy Asthma Immunol..

[12] Yang E.J., Beck K.M., Sekhon S., Bhutani T., Koo J. (2019). The impact of pediatric atopic dermatitis on families: A review. Pediatr. Dermatol..

[13] Barbarot S., Silverberg J.I., Gadkari A., Simpson E.L., Weidinger S., Mina-Osorio P., Rossi A.B., Brignoli L., Mnif T., Guillemin I. (2022). The family impact of atopic dermatitis in the pediatric population: Results from an international cross-sectional study. J. Pediatr..

[14] Huang E., Ong P.Y. (2018). Severe atopic dermatitis in children. Curr. Allergy Asthma Rep..

[15] Capozza K., Schwartz A., Lang J.E., Chalmers J., Camilo J., Abuabara K., Kelley K., Harrison J., Vastrup A., Stancavich L. (2022). Impact of childhood atopic dermatitis on life decisions for caregivers and families. J. Eur. Acad. Dermatol. Venereol..

[16] Wadonda-Kabondo N., Sterne J.A., Golding J., Kennedy C.T., Archer C.B., Dunnill M.G., Team A.S. (2004). Association of parental eczema, hayfever, and asthma with atopic dermatitis in infancy: Birth cohort study. Arch. Dis. Child..

[17] Kantor R., Kim A., Thyssen J.P., Silverberg J.I. (2016). Association of atopic dermatitis with smoking: A systematic review and meta-analysis. J. Am. Acad. Dermatol..

[18] AlShatti K.A., Ziyab A.H. (2020). Pet-keeping in relation to asthma, rhinitis, and eczema symptoms among adolescents in Kuwait: A cross-sectional study. Front. Pediatr..

[19] Silverberg J.I., Barbarot S., Gadkari A., Simpson E.L., Weidinger S., Mina-Osorio P., Rossi A.B., Brignoli L., Saba G., Guillemin I. (2021). Atopic dermatitis in the pediatric population: A cross-sectional, international epidemiologic study. Ann. Allergy Asthma Immunol..

[20] Asher M.I., Keil U., Anderson H.R., Beasley R., Crane J., Martinez F., Mitchell E.A., Pearce N., Sibbald B., Stewart A.W. (1995). International Study of Asthma and Allergies in Childhood (ISAAC): Rationale and methods. Eur. Respir. J..

[21] Charman C.R., Venn A.J., Williams H.C. (2004). The patient-oriented eczema measure: Development and initial validation of a new tool for measuring atopic eczema severity from the patients’ perspective. Arch. Dermatol..

[22] Charman C.R., Venn A.J., Ravenscroft J.C., Williams H.C. (2013). Translating Patient-Oriented Eczema Measure (POEM) scores into clinical practice by suggesting severity strata derived using anchor-based methods. Br. J. Dermatol..

[23] Silverberg J.I., Chiesa Fuxench Z.C., Gelfand J.M., Margolis D.J., Boguniewicz M., Fonacier L., Grayson M.H., Simpson E.L., Ong P.Y. (2018). Content and construct validity, predictors, and distribution of self-reported atopic dermatitis severity in US adults. Ann. Allergy Asthma Immunol..

[24] Lawson V., Lewis-Jones M.S., Finlay A.Y., Reid P., Owens R.G. (1998). The family impact of childhood atopic dermatitis: The dermatitis family impact questionnaire. Br. J. Dermatol..

[25] Ramirez F.D., Chen S., Langan S.M., Prather A.A., McCulloch C.E., Kidd S.A., Cabana M.D., Chren M.M., Abuabara K. (2019). Assessment of sleep disturbances and exhaustion in mothers of children with atopic dermatitis. JAMA Dermatol..

[26] Cheng B.T., Silverberg J.I. (2021). Association of pediatric atopic dermatitis and psoriasis with school absenteeism and parental work absenteeism: A cross-sectional United States population-based study. J. Am. Acad. Dermatol..

[27] Yamamoto-Hanada K., Yang L., Ishitsuka K., Ayabe T., Mezawa H., Konishi M., Shoda T., Matsumoto K., Saito H., Ohya Y. (2017). Allergic profiles of mothers and fathers in the Japan Environment and Children’s Study (JECS): A nationwide birth cohort study. World Allergy Organ. J..

[28] Shinohara M., Matsumoto K. (2017). Fetal tobacco smoke exposure in the third trimester of pregnancy is associated with atopic eczema/dermatitis syndrome in infancy. Pediatr. Allergy Immunol. Pulmonol..

[29] Sugiyama T., Sugiyama K., Toda M., Yukawa T., Makino S., Fukuda T. (2002). Risk factors for asthma allergic diseases among 13–14-year-old school children in Japan. Allergol. Int..

[30] Tanaka K., Miyake Y., Furukawa S., Arakawa M. (2017). Pre- and postnatal smoking exposure and risk of atopic eczema in young Japanese children: A prospective prebirth cohort study. Nicotine Tob. Res..

[31] Jing D., Li J., Tao J., Wang X., Shan S., Kang X., Wu B., Zhang Y., Xiao Y., Chen X. (2020). Associations of second-hand smoke exposure with hand eczema and atopic dermatitis among college students in China. Sci. Rep..

[32] Kim S.Y., Sim S., Choi H.G. (2017). Atopic dermatitis is associated with active and passive cigarette smoking in adolescents. PLoS ONE.

[33] Lee A., Lee S.Y., Lee K.S. (2020). Association of secondhand smoke exposure with allergic multimorbidity in Korean adolescents. Sci. Rep..

[34] Miyake Y., Ohya Y., Tanaka K., Yokoyama T., Sasaki S., Fukushima W., Ohfuji S., Saito K., Kiyohara C., Hirota Y. (2007). Home environment and suspected atopic eczema in Japanese infants: The Osaka maternal and child health study. Pediatr. Allergy Immunol..

[35] Kurosaka F., Nakatani Y., Terada T., Tanaka A., Ikeuchi H., Hayakawa A., Konohana A., Oota K., Nishio H. (2006). Current cat ownership may be associated with the lower prevalence of atopic dermatitis, allergic rhinitis, and Japanese cedar pollinosis in schoolchildren in Himeji, Japan. Pediatr. Allergy Immunol..

[36] Katoh N., Ohya Y., Ikeda M., Ebihara T., Katayama I., Saeki H., Shimojo N., Tanaka A., Nakahara T., Nagao M. (2020). Japanese guidelines for atopic dermatitis 2020. Allergol. Int..

[37] Furue M., Hashimoto-Hachiya A., Tsuji G. (2019). Aryl hydrocarbon receptor in atopic dermatitis and psoriasis. Int. J. Mol. Sci..

[38] Napolitano M., Fabbrocini G., Martora F., Picone V., Morelli P., Patruno C. (2021). Role of aryl hydrocarbon receptor activation in inflammatory chronic skin diseases. Cells.

