# Supplementary material for: Impact of the Family and Household Environment on Pediatric Atopic Dermatitis in Japan
Source: J Clin Med. 2023 Apr 20;12(8):2988. doi: 10.3390/jcm12082988 (PMC10145633; doi:10.3390/jcm12082988)

Impact of the family and household environment on pediatric atopic dermatitis in Japan

Supplementary Material

Hidehisa Saeki [1*], Yukihiro Ohya [2], Hisakatsu Nawata [3], Kazuhiko Arima [3], Miho Inukai [4], Ana B. Rossi [5] and Gaelle Bego-Le-Bagousse [6]

[1] Department of Dermatology, Nippon Medical School, 1-1-5 Sendagi, Bunkyo-ku, Tokyo, 113-8603, Japan
[2] Allergy Center, National Center for Child Health and Development, Setagaya-ku, 157-8535 Tokyo, Japan
[3] Specialty Care Medical, Sanofi K.K., Shinjuku-ku, 163-1488 Tokyo, Japan
[4] Market Access, Sanofi K.K., Shinjuku-ku, 163-1488 Tokyo, Japan
[5] Sanofi, Cambridge, 02139 MA, USA
[6] Sanofi, Chilly-Mazarin, 91380 Paris, France
* Correspondence: h-saeki@nms.ac.jp; Tel.: +81-3-3822-2131

Supplemental Figures

Supplemental Figure S1 Current residency of families of Japanese pediatric patients with atopic dermatitis by disease severity and age group.

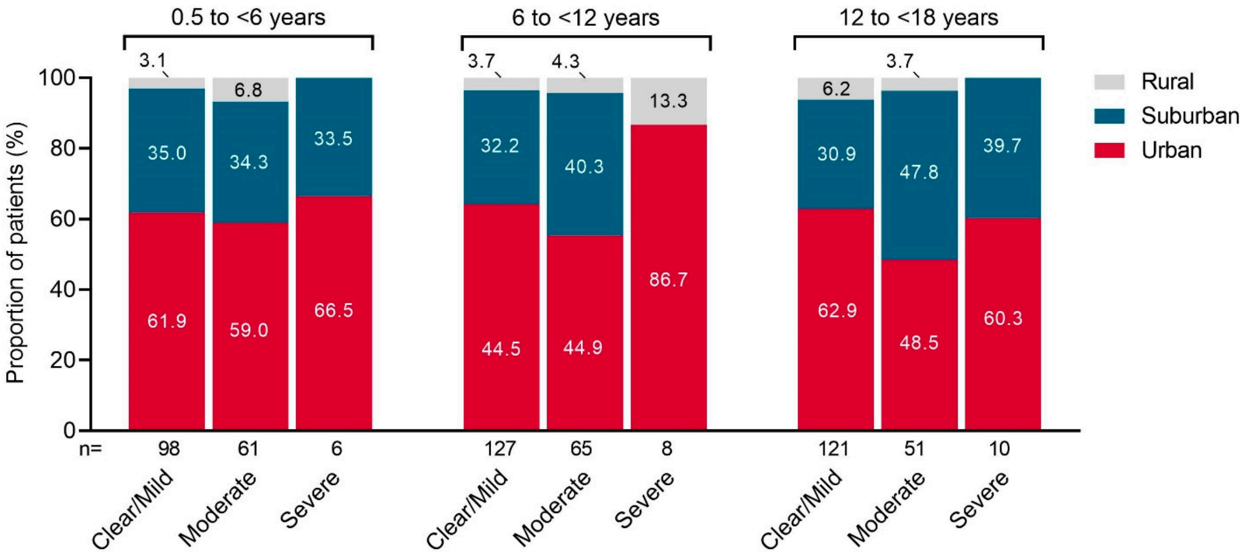

Supplemental Figure S2 Proportion of households with or without smokers by severity of atopic dermatitis in Japanese pediatric patients across age groups.

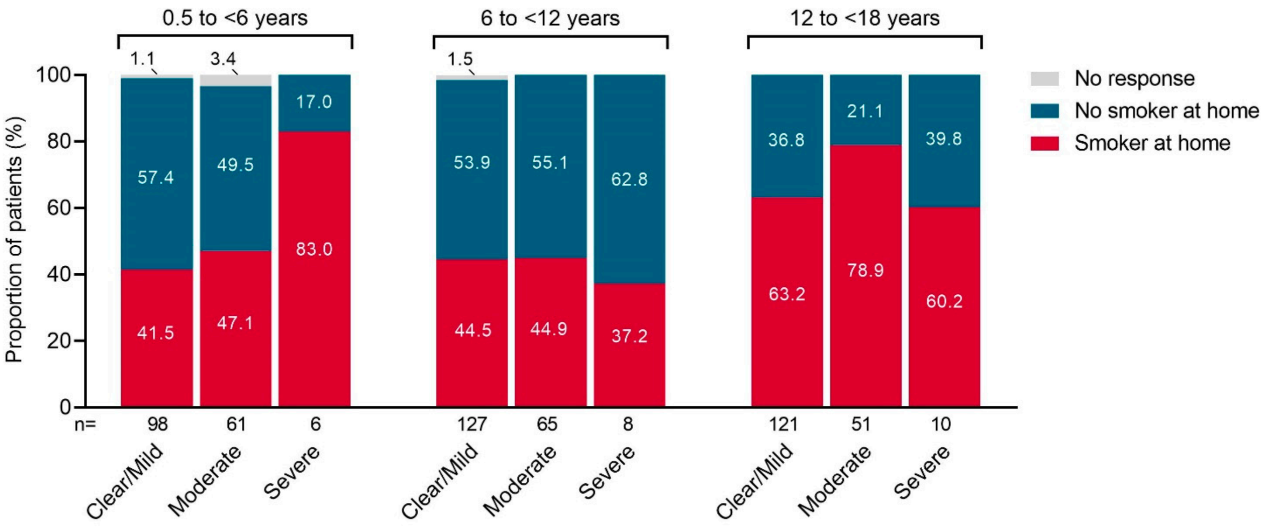

Supplemental Figure S3 Number of household pets by severity of atopic dermatitis in Japanese pediatric patients across age groups.

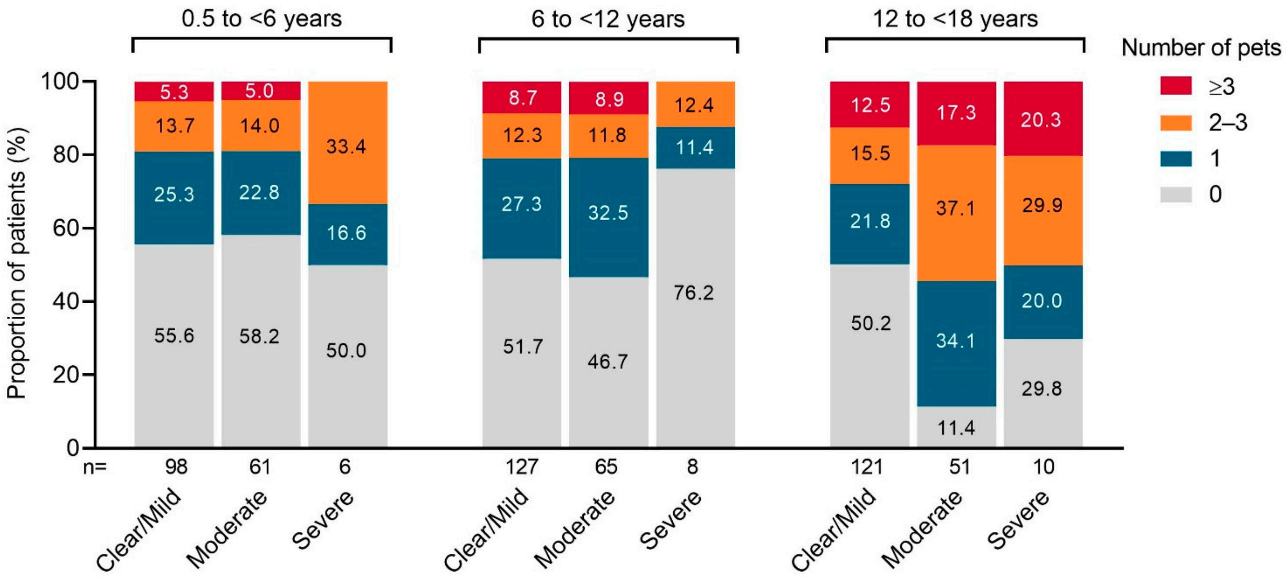

Supplemental Figure S4 Parents' highest education level by severity of atopic dermatitis in Japanese pediatric patients across age groups.

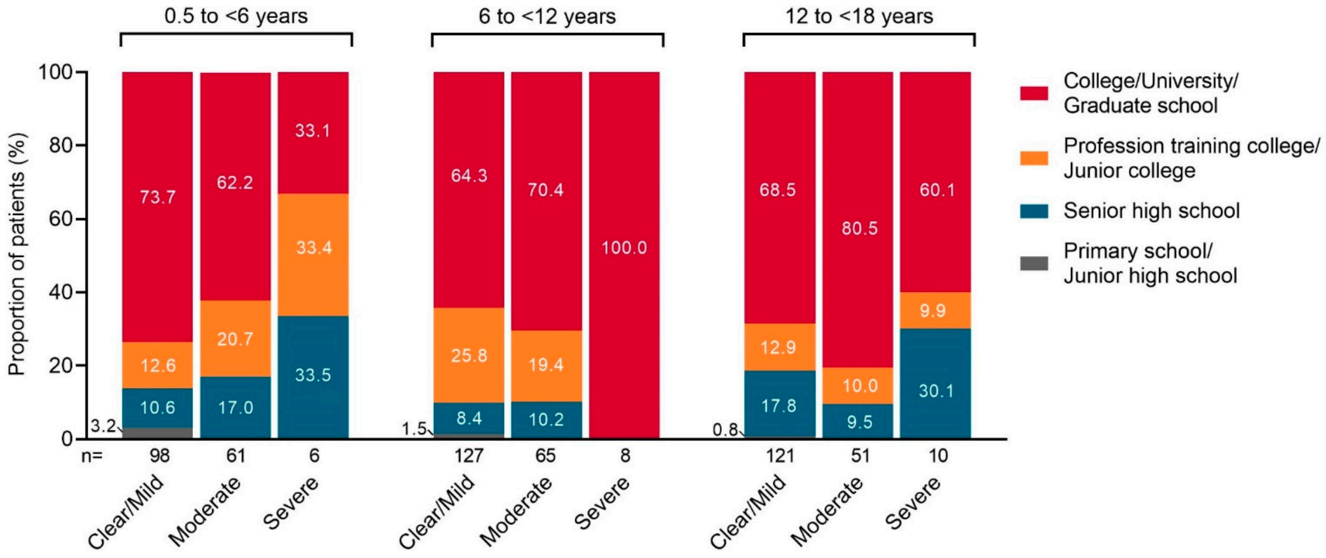

Supplemental Figure S5 Parents' employment status by severity of atopic dermatitis in Japanese pediatric patients across age groups.

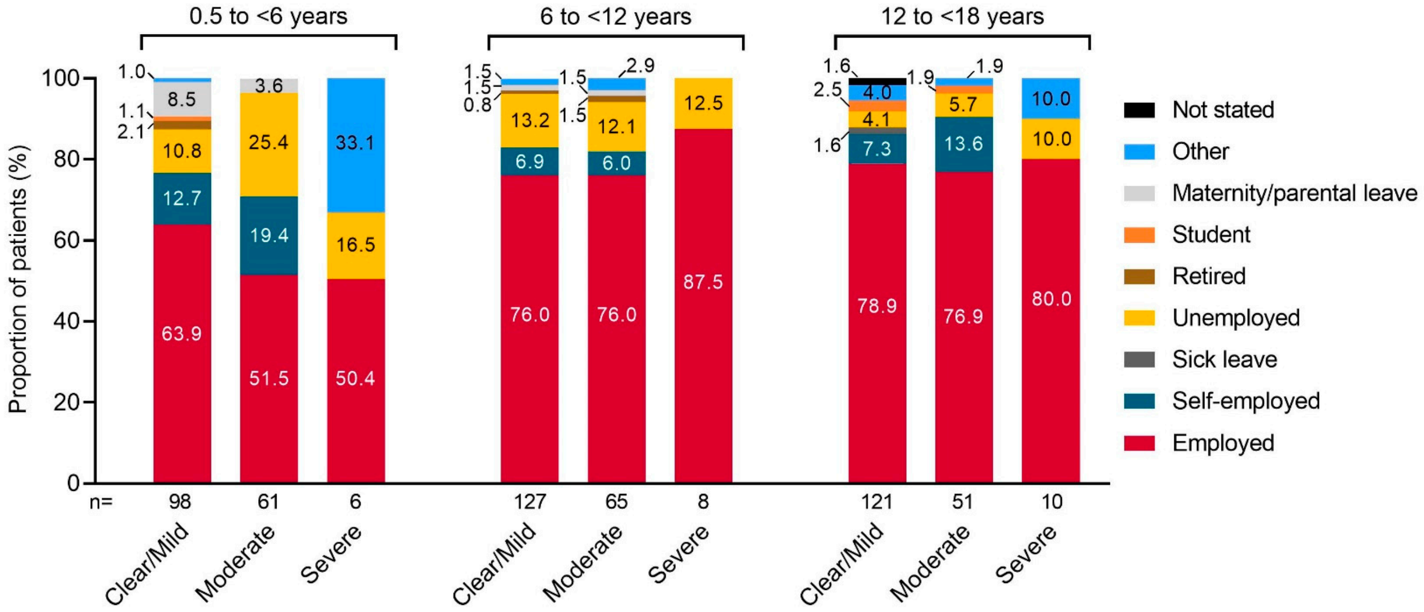

Supplement: Supplementary file 1 [file jcm-12-02988-s001.zip › jcm-2281018-supplementary.pdf]
